# Post-traumatic stress disorder in earthquake survivors living in temporary shelter areas in Hatay central districts: a cross-sectional study

**DOI:** 10.1186/s12888-025-06919-9

**Published:** 2025-05-07

**Authors:** Ertan Yilmaz, Mehmet Erdem

**Affiliations:** 1 Present Address: Department of Psychiatry, Hatay Mustafa Kemal University Tayfur Ata Sokmen Faculty of Medicine, Hatay, Turkey; 2 Department of Public Health, Hatay Mustafa Kemal University Tayfur Ata Sokmen Faculty of Medicine, Hatay, Turkey

**Keywords:** Post-Traumatic stress disorder, PTSD, Earthquake, Risk factors, Temporary shelter, Earthquake survivors

## Abstract

**Background:**

The 2023 Kahramanmaraş earthquakes resulted in extensive destruction, significant loss of life, and widespread displacement, with Hatay province being the most severely affected region. Earthquake survivors residing in temporary shelters face an elevated risk of long-term psychological consequences, particularly post-traumatic stress disorder (PTSD) and depression. This study aims to assess the prevalence of PTSD and depression among earthquake survivors in temporary shelter areas within the central districts of Hatay and to identify associated risk factors contributing to PTSD.

**Methods:**

This cross-sectional, population-based study was conducted among 400 adult earthquake survivors following the 2023 Kahramanmaraş earthquakes. Participants were selected using a multistage cluster sampling method. Data on participants’ sociodemographic characteristics, earthquake experiences, and psychiatric history were collected through self-report questionnaires. PTSD was assessed using the PTSD Checklist for DSM-5 (PCL-5), while depression was evaluated using the Beck Depression Inventory.

**Results:**

PTSD was identified in 29.0% of participants, while depression was observed in 38.8%. Key risk factors for PTSD included unemployment (OR = 2.590, *p* = 0.004), the loss of a family member (OR = 2.351, *p* = 0.017), a pre-existing psychiatric diagnosis prior to the earthquakes (OR = 2.245, *p* = 0.007), alcohol use (OR = 2.310, *p* = 0.019), smoking (OR = 1.663, *p* = 0.044), and experiencing a high level of fear during the earthquakes (OR = 2.151, *p* = 0.002).

**Conclusions:**

These findings highlight the critical need for large-scale psychosocial support and intervention programs in the post-disaster period. Identifying risk factors for PTSD may aid in the development of targeted treatment strategies and preventive interventions for affected individuals.

## Background

Earthquakes, classified as natural disasters, are recognized as traumatic events due to their widespread impact, causing loss of life and destruction on a large scale. A key distinction between earthquakes and other disasters is that affected individuals must continue to live with the persistent risk of recurrence [1].

Earthquakes can have significant consequences for both physical and psychological health. Post-disaster traumatic stress reactions encompass a broad spectrum, ranging from grief, fear, and anxiety to more severe psychiatric conditions, such as post-traumatic stress disorder (PTSD). Among the psychiatric disorders that may arise following traumatic events, PTSD is one of the most extensively studied and most prevalent [2]. According to the DSM-5 criteria, exposure to a traumatic event, which is a key diagnostic criterion for PTSD, may occur through direct experience or witnessing actual or threatened events involving death, serious injury, or sexual violence. PTSD is characterized by multiple symptom clusters, including intrusion, avoidance, negative alterations in cognition and mood, and changes in arousal and reactivity [3].

Research on PTSD has demonstrated that the prevalence of PTSD among disaster survivors ranges from 5 to 60% [4], which is significantly higher than its prevalence in the general population, ranging from 0 to 3.8% [5].

One of the major challenges posed by disasters is the potential permanence of psychological effects in some survivors. According to the National Comorbidity Survey, one-third of disaster survivors continue to experience PTSD symptoms even a decade after the traumatic event [6]. In a 25-year follow-up study, Goenjian et al. highlighted that PTSD and depression became chronic in most survivors of the Armenian earthquake; however, they noted that early psychological interventions could have lasting positive effects [7]. Similarly, a study conducted three years after the Nepal earthquake found PTSD in approximately one-fifth of participants [8]. Zhang et al. reported a PTSD prevalence of 10.3% three years post-Wenchuan earthquake in China [9].

Various risk factors encountered before, during, and after a disaster can contribute to the development of psychopathology in its aftermath. Pre-disaster risk factors include age, gender, education level, pre-existing psychiatric disorders, and adverse childhood experiences. Risk factors during the disaster encompass proximity to the earthquake epicenter, trauma severity, and the loss of a loved one. Post-disaster risk factors involve the loss of social support and ongoing life stressors [10, 11].

Among the Kahramanmaraş earthquakes, the epicenter of the first, with a magnitude of 7.7 on the Richter scale, was in the Pazarcık district of Turkey’s Kahramanmaraş province. This earthquake occurred at 04:17 on February 6, 2023. The epicenter of the second earthquake, which had a magnitude of 7.6, was in the Elbistan district of the same province and occurred at 13:30 on the same day [12]. These two earthquakes affected a vast area encompassing 11 provinces, including Kahramanmaraş and Hatay, with the total population of these regions accounting for approximately 16% of Turkey’s population [13].

The World Health Organization (WHO) classified this disaster as a **“**Level 3 Emergency,” the highest level of emergency response [14]. Following the initial earthquakes, a third earthquake, with a magnitude of 6.4, struck the Yayladağı district of Hatay province at 20:04 on February 20, 2023. Since the first earthquake, more than 33,000 aftershocks have been recorded as of May 6, 2023.

According to official reports, the earthquakes resulted in over 50,000 fatalities and more than 100,000 injuries. The number of collapsed and severely damaged buildings is estimated at approximately 500,000. The economic cost of this disaster to Turkey is estimated to be approximately 85 billion United States (US) dollars [13].

Hatay province experienced the highest level of destruction and loss of life from these earthquakes. Notably, 40% of all earthquake-related fatalities occurred in Hatay [15]. Consequently, the sample for this study was selected from individuals residing in temporary shelter areas within the central districts of Hatay, namely Antakya and Defne.

To the best of our knowledge, no population-based study has yet examined the mental health outcomes of adult earthquake survivors in Hatay province and the associated risk factors. Based on official data regarding these earthquakes, we hypothesized that residents of Hatay’s central districts experienced significant exposure to earthquake-induced trauma, leading to a high prevalence of PTSD and depression in these areas.

To evaluate this hypothesis, the primary outcome of this study was defined as the prevalence of PTSD and depression among individuals living in temporary shelter areas within the central districts of Hatay. The secondary outcome was identified as the risk factors contributing to these conditions. Identifying these factors may facilitate in the development of targeted psychosocial support programs and appropriate treatment interventions for earthquake survivors.

## Materials and methods

### Study design and setting

This study was designed as a cross-sectional, population-based study. Ethical approval was obtained from the Ethics Committee of Hatay Mustafa Kemal University in accordance with the Declaration of Helsinki (Approval Date: 29.08.2023, Approval No: 26). The study was conducted in the central districts of Hatay, specifically Antakya and Defne, during October and November following the earthquakes.

### Population and sample

The research was conducted in a single city. Clusters for the research sample were selected from both central districts, Antakya and Defne, located in the heart of Hatay, the city most severely affected by the earthquake and where the greatest destruction occurred. The study population consisted of adults living in container cities within the Antakya and Defne districts of Hatay province. According to municipal authorities, as of the study period, approximately 70,000 individuals were living in 48 container cities in Antakya, while approximately 10,000 individuals were residing in 18 container cities in Defne, totaling around 80,000 people in temporary shelter areas in the central districts of Hatay. The expected incidence was estimated at 50% based on previous studies, with a target sample size of at least 384 participants, calculated using a 5% margin of error and a 95% confidence interval [16]. Multistage cluster sampling was employed to select participants for the study. Container cities with a population of fewer than 1,000 residents, as well as those housing officials from outside the province who had not directly experienced the earthquakes, were excluded from the sampling frame.

Consequently, 10 individuals were randomly selected from each of the 40 container cities with 30 located in Antakya and 10 in Defne, using simple random sampling. Thus, the final study sample comprised a total of 400 participants across these 40 groups.

Individuals who were unavailable for interviews during the day due to work commitments were approached again in the evening. If participants could not be reached in both attempts or declined to participate in the study, replacement participants were selected from neighboring containers in equal numbers to maintain the sample size.

### Study inclusion criteria

1. Being an earthquake survivor.
2. Being over 18 years old.
3. Living in temporary shelter.
4. Holding citizenship of the Republic of Turkey.
5. Having the cognitive capacity to understand and answer the questionnaire.
6. Having volunteered to participate in the study.
7. *Exclusion criteria*

Residing in permanent residences.

Residing outside the borders of Antakya and Defne districts.

### Data collection

Prior to data collection, a pilot study was conducted with 10 participants as part of the training provided to the interviewers by the researchers. Study data were collected using self-report questionnaires completed by participants with assistance from two university graduate interviewers, one male and one female. For participants who had difficulty reading the questionnaire, interviewers read the questions aloud. Before administering the questionnaire, the interviewers informed participants about the study’s objectives and the questionnaire’s content. Written and verbal consent was obtained prior to participation. The questionnaire was administered either in the participants’ containers or in other preferred locations near their residences. Completion of the questionnaire took approximately 45–60 min. Participants did not receive any financial compensation for their participation in the study.

### Measures

The questionnaire, developed by the researchers, comprised 38 questions assessing participants’ sociodemographic characteristics (age, gender, education level, income level) and adverse events experienced before, during, and after the earthquakes.

### Post-traumatic stress disorder checklist for DSM-5 (PCL-5)

The PTSD Checklist for the Diagnostic and Statistical Manual of Mental Disorders, Fifth Edition (DSM-5) (PCL-5), is a 20-item self-report instrument developed in accordance with DSM-5 criteria for PTSD. It assesses PTSD symptoms, which are categorized into four symptom clusters reflecting the DSM-5 diagnostic criteria: reexperiencing, avoidance, negative alterations in cognition and mood, and hyperarousal. Each item is scored on a scale from 0 to 4, with a maximum possible total score of 80 points [17]. The validity and reliability of the Turkish version of the PCL-5 were established by Boysan et al. [18]. Their study determined that a total PCL-5 score of 47 or higher was the optimal cutoff value for predicting PTSD, with a sensitivity of 82% and a specificity of 71%.

### Beck depression inventory (BDI)

The Beck Depression Inventory (BDI), developed by Beck et al., is a 21-item self-report instrument designed to assess the severity of depression in individuals using a 4-point Likert scale [19]. Each item is scored between 0 and 3, with a maximum possible total score of 63 points. The validity and reliability of the Turkish version of the BDI were established by Hisli, who reported a Cronbach’s alpha coefficient of 0.80 [20].

### Statistical analysis

Statistical analyses were conducted using SPSS 21.0 (Statistical Product and Service Solutions for Windows, Version 21.0, IBM Corp., Armonk, NY, USA, 2012).

Descriptive statistics, including row percentages, means, and standard deviations, were used to summarize the data. Pearson’s chi-square test and the chi-square test with Yates’ continuity correction were applied to compare two or more groups in terms of categorical variables. Student’s *t*-test was used to assess the relationship between two continuous variables and the dependent variable. Independent variables that exhibited significant associations in binary analyses were included in a backward logistic regression analysis to identify the factors that predict the dependent variable. A probability (*p*) value of ≤ 0.05 was considered statistically significant.

## Results

The final study sample comprised 400 individuals, replacing the 83 individuals (17.2%) who declined participation with an equal number of residents from neighboring containers. The mean age of the sample was 38.0 ± 14.4 years, with 53% female and 47% male participants. Participants’ sociodemographic characteristics are summarized in Table 1.

**Table 1:** The relationships between participants’ sociodemographic characteristics and the development of PTSD

Variables		Overall sample	PTSD, no	PTSD, yes	p-value **
		%	%*	%*	
Gender	Female	53.0	66.0	34.0	**0,020**
	Male	47.0	76.6	23.4	
Marital status	Single	19.3	75.3	24.7	0,473
	Married	64.8	72.2	27.8	
	Widowed/divorced/spouse deceased	16.0	60.9	39.1	
Education level	Primary education or below	53.0	68.4	31.6	0,223
	Secondary education or above	47.0	73.9	26.1	
Employment status	Unemployed	74.5	66.8	33.2	**0,001**
	Employed	25.5	83.3	16.7	
Lives alone or with family	Alone	8.5	70.6	29.4	0,956^a^
	With family	91.5	71.0	29.0	
Smoking status	Smoker	51.0	63.7	36.3	**0,001**
	Nonsmoker	49.0	78.6	21.4	
Alcohol use status	Drinker	12.3	53.1	46.9	**0,005** ^**a**^
	Nondrinker	87.8	73.5	26.5	
		**Mean ± SD**	**Mean ± SD**	**Mean ± SD**	
Age		38.0 ± 14.4	36.6 ± 14.5	36.6 ± 13.9	0.202^b^

*Row Percentage, ** Pearson’sChi-Square test, ^a^Chi-square test with Yates continuity correction, ^b^Student’s t-test,

SD; Standard Deviation

According to PCL-5 scores, 29.0% of participants met the criteria for PTSD, while 38.8% were diagnosed with depression based on BDI scores. PTSD prevalence was higher among female participants (34.4%) compared to male participants (23.4%) (*p* = 0.020). Additionally, PTSD was diagnosed in 74.5% of unemployed individuals compared to 25.5% of employed individuals (*p* = 0.001), 36.3% of smokers compared to 21.4% of non-smokers (*p* = 0.001), and 87.8% of alcohol users compared to 12.3% of non-drinkers (*p* = 0.005) (Table 1). The associations between participants’ experiences before, during, and after the earthquakes and the development of PTSD are summarized in Table 2. The mean fear score during the earthquakes was significantly higher among individuals diagnosed with PTSD (8.1 ± 2.5) compared to those without a PTSD diagnosis (6.8 ± 2.9) (*p* < 0.001). Similarly, the mean total BDI scores were 23.7 ± 9.8 for individuals with PTSD and 11.7 ± 7.5 for those without PTSD (*p* < 0.001).

**Table 2:** The relationships between the events that the participants experienced before, during and after the earthquakes and the development of PTSD

Variables	Overall Sample (%)	PTSD, no (%)	PTSD, yes (%)	p-value
**Previous psychiatric disorder**				
No	83	75.0	25.0	< 0.001
Yes	17	51.5	48.5	
**Familial history of psychiatric disorder**				
No	88	72.3	27.3	0.058^a^
Yes	12	58.3	41.7	
**Trapped under rubble**				
No	88.8	72.4	27.6	0.121^a^
Yes	11.3	60	40	
**Physical injuries**				
No	69.8	75.3	24.7	0.004
Yes	30.3	61.2	38.8	
**Waiting for relatives to be rescued from the rubble**				
No	50.3	77.6	22.4	0.003
Yes	49.8	64.3	35.7	
**Participated in rescue efforts**				
No	82.3	70.5	29.5	0.753^a^
Yes	17.8	73.2	26.8	
**Damage to the house**				
Destroyed in major and aftershock earthquakes	25.8	63.1	36.9	0.040
Not destroyed (heavily, moderately or slightly damaged or undamaged)	74.3	73.7	26.3	
**Lost a member of the family**				
No	88	73.6	26.4	0.004^a^
Yes	12	52.1	47.9	
**Lost relatives**				
No	23	73.0	27.0	0.933
Yes	77	71.1	28.9	
**Lost a close friend**				
No	32.5	80.0	20.0	0.006
Yes	67.5	66.7	33.3	
**Place of residence after the earthquakes**				
Always lived in Hatay	28.5	74.6	25.4	0.322
Lived outside Hatay for a while	71.5	69.6	30.4	
**Loss of income**				
No	15.5	85.5	14.5	0.010^a^
Yes	84.5	68.3	31.7	
**Psychiatric disorder after the earthquakes**				
No	90.8	73.3	26.7	0.003^a^
Yes	9.3	48.6	51.4	
	**Mean ± SD**	**Mean ± SD**	**Mean ± SD**	**p-value**
**Fear level (between 0 and 10)**	7.2 ± 2.9	6.8 ± 2.9	8.1 ± 2.5	< 0.001^b^
**Total PCL-5 score**	36.3 ± 15.9	28.5 ± 11.1	55.4 ± 16.9	< 0.001^b^
**Total BDI score**	15.2 ± 9.9	11.7 ± 7.5	23.7 ± 9.8	< 0.001^b^

*Row Percentage, ** Pearson’sChi-Square test, ^a^Chi-square test with Yates continuity correction, ^b^Student’s t-test, SD; Standard Deviation

The fear level was measured on a scale from 0 to 10. It was analyzed as a categorical variable, with the median fear score of 8 designated as the cutoff value for predicting PTSD.

Backward logistic regression analysis was conducted for 14 binary independent variables with a *p*-value of less than 0.10, considering PTSD development as the dependent variable. The *R²* value of the final model was calculated as 0.232 (*p* < 0.001) (Table 3).

**Table 3:** Risk factors for PTSD revealed by backward logistic regression analysis

Predictors	B	p-value	OR	%95 CI
Unemployed	0.952	0.004	2.590	1.358–4.937
Lost a member of the family	0.855	0.017	2.351	1.167–4.737
Alcohol use	0.837	0.019	2.310	1.147–4.650
Previous psychiatric disorder	0.809	0.007	2.245	1.253–4.025
Fear score	0.766	0.002	2.151	1.326–3.489
Lost a close friend	0.634	0.024	1.885	1.087–3.266
Smoking	0.509	0.044	1.663	1.013–2.730

OR = Odds Ratio, CI = Confidence Interval

According to the model, unemployed individuals had a 2.590-fold higher risk of developing PTSD. Those who lost a family member due to the earthquakes had a 2.351-fold higher risk, alcohol users had a 2.310-fold higher risk, individuals with a pre-existing psychiatric disorder had a 2.245-fold higher risk, those with a fear score of 9 or above had a 2.151-fold higher risk, individuals who experienced the loss of a close friend due to the earthquakes had a 1.884-fold higher risk, and smokers had a 1.663-fold higher risk of developing PTSD.

## Discussion

This study demonstrated that the higher prevalence of PTSD and depression remained notably high among earthquake survivors residing in temporary shelter areas in Hatay city center approximately one year after the 2023 Kahramanmaraş earthquakes. PTSD was identified in 29.0% of participants, while depression was observed in 38.8%. Studies conducted shortly after an earthquake typically report a higher prevalence of PTSD.

Early studies on the Kahramanmaraş earthquakes reported PTSD prevalence rates ranging from 54.1 to 72.5% [21, 22]. Similarly, in a study conducted in the early post-disaster period in the Gölcük district of Kocaeli province, the epicenter of the Marmara earthquake, Başoğlu et al. identified a PTSD prevalence of 43% [23]. Following the Wenchuan earthquake in China, PTSD was detected in approximately two-thirds of participants in a study conducted within the first month [24]. However, studies conducted one year later reported PTSD prevalence rates between 21.5% and 41% [25], which aligns with the findings of this study.

A meta-analysis conducted by Dai et al. found that studies conducted within the first nine months following an earthquake report a higher incidence of PTSD compared to those conducted afterward [26]. The severity of trauma and proximity to the earthquake’s epicenter are critical factors influencing PTSD development [27, 28].

The February earthquake was one of the most devastating in the country’s history. Given that the study was conducted in the region experiencing the highest levels of destruction and fatalities, the observed PTSD prevalence rates are not unexpected.

In a study conducted by Kilpatrick et al., the proportion of adults in the United States who had experienced trauma during their lifetime was reported as 89.7%, while the lifetime prevalence of PTSD was 8.3% [29]. These findings highlight the importance of identifying additional factors contributing to PTSD, as trauma exposure alone is insufficient for its development. In this context, one of the key objectives of this study was to determine the predictors of PTSD.

Multivariate analysis further identified unemployment after the earthquakes, fear experienced during the earthquakes, the loss of a family member due to the earthquakes, the loss of a close friend due to the earthquakes, alcohol use, smoking, and a pre-existing psychiatric disorder as significant predictors of PTSD.

Unemployment following the earthquakes emerged as the most significant risk factor for PTSD development. Unemployment may contribute to PTSD development through both financial instability and the loss of a social environment. Notably, unemployment was prevalent within the study population. The data collection period faced with a challenging phase in which temporary accommodation centers provided shelter, yet employment opportunities had not been fully reestablished. The high unemployment rate serves as a critical indicator of the disaster’s severity.

Numerous studies conducted after other earthquakes have consistently demonstrated an association between financial losses, loss of regular income, and PTSD [16, 30, 31]. Furthermore, a 5.5-year follow-up study conducted after the 2011 Japan earthquake identified a strong relationship between job loss and persistent PTSD. Additionally, subjective economic losses were associated with the delayed onset of PTSD in the same study [32]. In a study examining the relationship between unemployment and PTSD following the Chilean earthquake, it was proposed that the development of PTSD could help explain unemployment through an alternative model. The study also hypothesized a potential reciprocal interaction among stress, unemployment, and psychological outcomes, suggesting the emergence of a bidirectional relationship [33].

Another significant predictor of PTSD was the level of fear experienced during the earthquakes. This finding aligns with previous studies conducted following earthquakes [34–38] and highlights the role of subjective trauma experiences in PTSD development. An increased perception of threat from the traumatic event, coupled with low controllability of stressors, further elevates the likelihood of traumatic stress occurring [39–42].

The amygdala plays a crucial role in threat perception and fear regulation. In a study involving individuals exposed to war trauma, Wingen et al. found that severe stress heightened the reactivity of the amygdala and insula. They also emphasized that individual differences in threat perception were associated with variability in amygdala activity regulation [43].

A study conducted with torture survivors demonstrated that prior preparation for trauma can mitigate symptoms of traumatic stress and general psychopathology. However, natural disasters such as earthquakes are unpredictable events for which individuals cannot prepare, and they are often followed by aftershocks. Such disasters can disrupt an individual’s fundamental belief that the world is structured, meaningful, and safe, thereby contributing to the development of traumatic stress [44].

Several studies conducted following the Marmara earthquake have highlighted that factors such as being inside a building during the earthquake and proximity to the earthquake’s epicenter were significant determinants of the level of fear experienced at the time of the event [35, 36]. The region where this study was conducted is located near the epicenters of the earthquakes, which occurred at a time when the majority of the population was indoors.

The loss of a family member or a close friend has been identified as a significant predictor of PTSD. Similarly, a meta-analysis by Tang et al. found that bereavement significantly increases the risk of developing PTSD [37]. Furthermore, a study conducted 10 years after the Wenchuan earthquake reported that the loss of close family members was a significant risk factor for the development of long-term PTSD [45]. This finding aligns with the well-documented observation that traumatic stress symptoms are triggered and sustained by adverse life events [38, 46].

However, several studies have suggested that bereavement is not a direct predictor of PTSD. Zhang emphasized that the nature of the relationship with the deceased, rather than the number of losses, plays a crucial role in the development of PTSD following an earthquake [47]. In a follow-up study conducted by Karamustafalıoğlu et al., while harm or loss affecting one’s relatives was a predictor of PTSD in the short term, this association was not observed ten years after the earthquake. The same study identified avoidance behavior as a predictor of PTSD [48]. Given that the region where this study was conducted has a cultural structure characterized by strong family ties, it is understandable that bereavement may contribute to PTSD development. The loss of a family member or close relative may have exacerbated the individual’s difficulty in processing the trauma by reducing the availability of essential social support needed for coping.

In addition to a history of alcohol use and smoking prior to the earthquakes, a pre-existing psychiatric disorder also emerged as a significant predictor of PTSD. Similarly, several studies have reported that prior trauma and pre-traumatic psychopathology are strong predictors of PTSD [46, 49, 50]. In a study conducted with disaster victims, North et al. found that while one-third of participants developed psychiatric disorders, only one-fifth developed a new psychiatric disorder following the disaster. This suggests that the majority of psychiatric diagnoses made after the disaster were pre-existing conditions [51]. However, this study did not assess traumatic experiences occurring before the earthquakes.

Several studies have demonstrated that individuals with a history of PTSD following previous traumatic experiences are at an increased risk of developing PTSD in response to subsequent traumatic events [52, 53]. In other words, the presence of prior trauma-induced psychopathology, rather than the traumatic experience itself, is a critical determinant of whether subsequent traumas will lead to psychopathology. Thus, individuals predisposed to pathological responses to stressors are more likely to develop PTSD following subsequent traumatic events.

Several studies have reported a high incidence of co-occurrence between PTSD and alcohol or tobacco use disorders [54]. Following the L’Aquila earthquake in Italy, an increase in alcohol and tobacco consumption was observed among individuals both with and without a psychiatric diagnosis with no reported decrease among any participants [55]. In contrast, a study conducted after the September 11 terrorist attacks in the United States observed a slight decrease in alcohol consumption, although other studies reported increases in both alcohol and tobacco use [56, 57].

The relationship between PTSD and substance use disorders is bidirectional; PTSD may contribute to increased alcohol consumption and smoking as coping mechanisms to alleviate its effects. Conversely, alcohol use and smoking can alter an individual’s stress response, heightening anxiety and arousal levels, thereby facilitating PTSD development [58]. Although participants in this study were asked about their alcohol and tobacco use, the presence of alcohol or tobacco use disorders and whether individuals had received treatment for such disorders were not assessed. Therefore, the findings on the association between PTSD and substance use should be interpreted with caution.

### Limitations

This study has several limitations. First, the prevalence of PTSD and depression was assessed using self-report questionnaires rather than clinical interviews. Second, interviewer assistance for participants who had difficulty reading the questionnaire may have introduced response bias. Third, the study sample included only individuals residing in container cities, excluding those living in their own homes, which may limit the generalizability of the findings to the broader population. Finally, the study focused solely on earthquake-related traumas, without evaluating potential pre-earthquake traumatic experiences. However, the study also has several strengths. It was designed as a population-based study with a robust sampling method and conducted in one of the regions most severely affected by the earthquakes, enhancing the reliability of its findings.

## Conclusions

This study found that approximately one-third of participants residing in temporary shelters one year after the 2023 Kahramanmaraş earthquakes diagnosed with PTSD, while more than one-third were diagnosed with depression. These findings highlights the critical need for large-scale and early psychosocial interventions in the post-disaster period. Several factors were identified as significant risk factors for PTSD, including unemployment following the earthquakes, a pre-existing psychiatric disorder, the loss of a family member or close friend due to the earthquakes, smoking, alcohol use, and the level of fear experienced during the earthquake. Based on these factors, high-risk groups for PTSD can be identified, enabling the development of effective preventive and treatment strategies. The social and economic instability experienced by earthquake survivors further underscores the importance of individualized mental health support.

Future longitudinal studies that incorporate the impact of pre-earthquake traumatic experiences may offer a more comprehensive understanding of the development and trajectory of PTSD. Continuous monitoring of mental health in disaster-affected regions is essential for establishing sustainable, long-term mental health strategies.

## Data Availability

The data that support the findings of this study are not openly available due to reasons of sensitivity and are available from the corresponding author upon reasonable request.
